# Vortioxetine usage in an elderly patient with major depressive disorder and accompanied by multiple physical conditions: A case report

**DOI:** 10.1002/agm2.12268

**Published:** 2023-09-12

**Authors:** Xiaoman Wang, Xiaoqian Ma, Yicheng Long, Guowei Wu

**Affiliations:** ^1^ XiangYa School of Medicine Central South University Changsha China; ^2^ Department of Psychiatry, The Second Xiangya Hospital, National Clinical Research Center for Mental Disorders Central South University Changsha China

**Keywords:** chronic physical conditions, major depressive disorder, vortioxetine

## Abstract

Elderly patients with depressive disorder always have complex and diverse symptoms, and are mostly combined with chronic physical conditions. This case report presents a case of vortioxetine usage in a 67‐year‐old male patient with major depressive disorder and accompanied by multiple physical conditions.

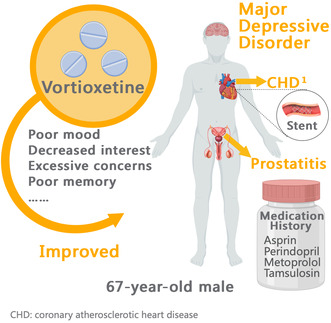

## 1 INTRODUCTION

In elderly people, depression mainly affects those with chronic physical diseases. Such as coronary heart disease, hypertension and diabetes. Vortioxetine is a novel antidepressant with multimodal activity that has two different types of pharmacologic targets: serotonin receptors and transporters that have been approved for the treatment of major depressive disorder (MDD).^1^ Both short‐term and long‐term clinical trials indicated that vortioxetine is effective and safe in patients with MDD. Other effects include improved cognitive function and quality of life in patients with MDD.^2^

However, no studies have reported the efficacy of vortioxetine in elderly patients with depression combined with chronic physical diseases so far. We hope that this case can provide some references for the use of antidepressants in the clinical practice.

## 2 CASE REPORT

The patient, a 67‐year‐old male, retired worker, presented with gradual poor mood, decreased interest, and excessive concerns for more than 3 years, and was admitted to the psychiatry department of the second Xiangya hospital on February 24, 2021. The patient reported that following the placement of a heart stent in September 2018, he experienced a gradual onset of symptoms including poor mood, listlessness, decreased interest, hypologia, hypokinesia, preference for solitude, excessive concerns about health, and emotional instability. Besides, the patient often complained of dizziness and poor memory. At first, his self‐care ability was fine, thus, his family did not pay attention to him, regarding his conditions as “a bad mood”. Later, he often had negative words and felt that it was meaningless to live. He first went to outpatient clinic in July 2020, and was considered the diagnosis of “depressive state.” He was recommended to take 10 mg qd of escitalopram and 0.5 mg bid of lorazepam. In further consultation in August, he claimed that his mood had improved, but decrease in interest remained significant and he did not care for housework. According to the doctor's advice, escitalopram tablets were increased to 15 mg qd and lorazepam tablets 0.5 mg bid. Since then the patient adhered to medication, but symptoms remained unstable. He switched to 1 tablet of flupentixol‐melitracen in September 2020, but the response was still not good. For further treatment, he sought hospitalization on February 24, 2021. Since the onset of the disease, his appetite was fair, and both urination and stool were normal.

The patient had a history of coronary atherosclerotic heart disease (CHD) and was implanted with a stent in September 2018. He had been taking aspirin enteric coated tablets 100 mg qd, perindopril tert butylamine tablets 4 mg qd, and metoprolol succinate tablets half qd and he had a history of “prostatitis” and had been taking tamsulosin hydrochloride sustained‐release capsule 0.2 mg qd. He denied other disease history, including hepatitis, tuberculosis, malaria, diabetes, cerebrovascular disease, and trauma. There was no similar history of mental diseases in his family history.

Physical examination: body temperature: 36.6°C, pulse: 97 times/min, respiration: 20 times/min, blood pressure: 146/92 mmHg. No obvious positive signs were found in the heart, lungs, abdomen, and nervous system.

Psychiatric examination: General condition: The patient was conscious and presented with passive performance, hypologia, lack of insight, and no disorientation. Cognitive activities: No signs of illusions, hallucinations, sensory disorders, and delusions. He had a poor short‐term memory and lacked concentration, while calculation ability and understanding were normal. Emotional activity: Coherent emotional response, constant depression, listlessness, no sense of pleasure, and self‐deprecating. No history of sustained hyperthymia. Volition activity: Hypobulia, unwilling to interact with others, preference for solitude, decreased interest. He denied suicidal thoughts, and had no action of impulse, self‐injuries, or suicide.

Auxiliary examination: There were no significant abnormalities in blood‐RT, urine‐RT, stool‐RT, liver and kidney function, fasting blood glucose, electrolytes, thyroid function, sex hormones, EEG, and chest X‐ray. Three pairs of hepatitis B, hepatitis C antigen, AIDS, and syphilis antibodies were all negative. Creatine kinase was measured at 34.7 U/L, showing a decrease, while BNP and high sensitivity troponin T showed no significant abnormalities. Triglyceride levels were elevated at 2.70 mmol/L, and high‐density lipoprotein cholesterol was decrease at 0.76 mmol/L. ECG: right axis deviation, counterclockwise rotation, prolonged QT interval (QT/QTc360/472 ms). Scale test: Hamilton Depression Scale (HAMD‐17 items): 26 points (severe depression); Hamilton Anxiety Scale (HAMA): 15 points (moderate anxiety).

Based on the characteristics of the patient's medical history and the evaluation results of the scale after admission, the patient met the Diagnostic and Statistical Manual of Mental Disorders, 5th Edition (DSM‐5) criteria for MDD. Mental disorders caused by physical diseases were ruled out for no significant abnormalities in his cardiac function and MRI were observed so far. Bipolar disorder was ruled out for no history of elation. Considering diagnosis: 1. MDD without psychotic symptoms; 2. CHD, postoperation of coronary stenting, NYHA II degrees; 3. Prostatitis. Given that the patient was an elderly male with multiple physical diseases, and currently had been taking four types of medication, lupenthixone melitracen was stopped after admission, and fluoxetine hydrobromide was dosed up from 5 mg to 10 mg qd within 2 days, combined with trazodone hydrochloride tablets 25–50 mg qn, and oxaloxetine that would be gradually decreased from 15 to 7.5 mg qn and would be stopped within 1–2 weeks to improve emotion and sleep. Meanwhile, psychotherapy and transcranial magnetic stimulation were arranged as auxiliary therapy. During hospitalization, no significant abnormalities were observed in blood pressure, blood glucose, and other monitoring results. After consultation with cardiovascular and urology departments, the original four drugs (aspirin enteric coated tablets 100 mg qd, perindopril tert butylamine tablets 4 mg qd, metoprolol succinate half tablet qd, and tamsulosin hydrochloride sustained‐release capsules 0.2 mg qd) were continued to be used as instructed for symptomatic treatment. The results of brain MRI showed right basal ganglia cavity infarction, white matter lesions (Fzekas1 grade), and brain atrophy. The results of electromyography examination showed abnormal sympathetic response of the foot skin. No special treatment was required after consultation with neurology department. According to the patient, there was no obvious discomfort after taking the medication, and his symptoms had improved significantly, including sense of pleasure, stable emotions, no obvious worries, and active communication with others. Volition activities were also increased, such as walking and exercising in the ward. During hospitalization, oxazepam was discontinued. HAMD and HAMA were retested on March 9, 2021 and scores were 6 and 5 points, respectively. The patient was discharged after improvement. Then, the patient adhered to taking medication and had regular further consultations. Afterwards, his emotions basically returned to normal levels. After follow‐up visits in 6 months, trazodone was gradually discontinued and fluoxetine hydrobromide was reduced to 5 mg qd. Fluoxetine hydrobromide was discontinued In March 2022. According to the follow‐up visits in June 2023, the patient's condition was stable, and he was able to live a normal life and take care of housework and children at home.

## 3 DISCUSSION

The symptoms of elderly patients with MDD are complex and diverse, usually accompanied by a variety of physical diseases including CHD, hypertension, diabetes; therefore, patients often take a variety of drugs. In clinical practice, it is necessary to ensure efficacy, safety, and tolerance, which increases the difficulty of treatment. Vortioxetine is a new multimodal antidepressant drug, which has shown good efficacy and high safety in clinical application since it was launched in China in 2018.^3^, ^4^

According to the second edition of the “Guidelines for the diagnosis and treatment of depressive disorders in China: The second edition” and authoritative guidelines such as CANMAT in Canada,^5^, ^6^ for patients with MDD, if the dosage of drug has been sufficient or has met the maximum tolerable dose of the individual treatment dose for more than 4 weeks, and the effect is still unsatisfactory, medication can be switched. If another antidepressant with a different mechanism still fails to act, drug combination is alternative. By comparing 21 antidepressants, previous studies suggested that vortioxetine was assured in efficacy, acceptability, and therapeutic effects on multiple symptoms of MDD including poor mood, anhedonia, decreased interest, physical discomfort, fatigue, and anxiety.^7^, ^8^, ^9^ More than half of patients who previously had poor effects on first‐line selective serotonin reuptake inhibitors (SSRIs) or serotonin norepinephrine dopamine reuptake inhibitors (SNRIs) antidepressant can achieve recovery standards after switching to vortioxetine.^10^ According to preclinical and clinical findings, compared to other antidepressants, vortioxetine functions as 5‐HT receptor modulator and SERT inhibitor, which results in the direct and indirect modulation of multiple neurotransmitter systems, and has effects in depression, and positive effects in cognitive dysfunction.^11^ And a pooled analysis suggests that vortioxetine is generally safe and well tolerated with few unexpected adverse events and has a similar effect on patients with MDD and comorbid cardiovascular disease or diabetes to that in the broader MDD population.^12^ In this case, with no significant improvement in depression and sleep of the patient after two rounds of sufficient course of therapy, the combination with other antidepressants was considered. According to the concept of whole course management, we need to concentrate on both the treatment of acute phase (the efficacy of vortioxetine in the acute phase is equivalent to that of venlafaxine^13^) and long‐term efficacy in elderly patients. Besides, original physical conditions of the patients, such as cardiovascular system and benign prostatic hyperplasia, were also essential.^14^ Studies have shown that vortioxetine has no significant effects on the central nervous system (CNS), cardiovascular system, metabolic endocrine system, and sexual function. However, gastrointestinal reactions such as nausea and vomiting may occur with a relatively high frequency especially during the first 1–2 weeks of medication, which is possibly relate to increased availability of 5‐HT in the gastrointestinal tract and CNS.^15^, ^16^, ^17^ In this case, considering SSRIs (escitalopram) and tricyclic antidepressants (flupentixol‐melitracen) had no obvious effects and suggestions given by guidelines, vortioxetine was chosen to improve depression as an alternative. In order to optimize the treatment plan, trazodone was combined to improve depression and sleep, and short‐term use of low‐dose oxazepam within 1–2 weeks was to alleviate anxiety and promote sleep. The above three drugs have no significant effects on metabolic indicators such as weight, blood lipids, and cardiovascular system.

Cognitive symptoms are the main residual symptoms after acute remission of depression.^18^ Improving cognitive function can promote functional recovery in patients with depression^14^; therefore, assisting patients to recover social function and improve quality of life has become the ultimate goal of treatment.^5^ The Canadian CANMAT Guidelines suggest that for patients with residual obvious cognitive symptoms after standardized antidepressant treatment, personalized treatment is needed to switch to a single antidepressant drug with a more accurate effect on improving cognitive symptoms, and vortioxetine is listed as class A recommended evidence.^6^ Through multiple studies, vortioxetine has been shown to enhance cognitive performance in various animal models and clinical trials, including executive function, attention, processing speed, learning and memory.^19^ The possible mechanism to explain the cognitive function effects of vortioxetine is the 5‐HT system, which is thought to be related in regulating prefrontal cortical circuits involved in cognitive processing.^20^ In this case, the patient consciously experienced a decrease in memory and attention since the onset of the disease, and had certain requirements for cognitive function. The patient reported that his memory and attention were significantly improved after treatment with vortioxetine. However, the limitation in this case is that cognitive function was not assessed during hospitalization; therefore, it is unable to compare the changes in cognitive function before and after treatment, which is yet to be discussed in further studies. At present, there is relatively little clinical experience in using vortioxetine for elderly patients with multiple physical diseases. Thus, when optimizing treatment plans for elderly depression patients, we recommend that clinicians balance the benefits and risks from multiple aspects, focus on evaluating the efficacy and interactions of the drugs, and strive to maximize the benefits.

## 4 CONCLUSIONS

To date, usage of vortioxetine in elderly patients with MDD accompanied by multiple physical conditions is exceptional. We acknowledge that the relationship between improvement of cognitive function and vortioxetine cannot be confirmed by this case. However, symptoms of the patient after medication supports this argument. We hope that this case can provide some references for the use of antidepressants in the clinical practice.

## AUTHOR CONTRIBUTIONS

Xiaoman Wang: idealization, assembly, and review of the literature and writing; Xiaoqian Ma and Yicheng Long: writing, revision, and editing; Guowei Wu: draft revision, writing, and supervision. All the authors agreed on the final text.

## FUNDING INFORMATION

Natural Science Foundation of Changsha City (kq2208322); College Students' Innovation and Entrepreneurship Training Program Project (2021105330303, XCX2022022).

## CONFLICT OF INTEREST STATEMENT

All authors declare that there is no conflict of interest in this study.

## CONSENT FOR PUBLICATION

Written informed consent was obtained from the patient for the publication of this case report.
